# Genome-wide association analysis of neutrophil granularity identifies CDK6 as a regulator of primary granules

**DOI:** 10.1016/j.isci.2025.113072

**Published:** 2025-07-07

**Authors:** Kathryn Fleming, Kate Burley, Fernando Ponce-Garcia, Parsa Akbari, Claire Naveh, Chris Rice, Przemysław Zakrzewski, Willem Gibbs, Sarah Groves, Drinalda Cela, Venizelos Papayannopoulos, Christopher J. Harbort, Andrew Mumford, Borko Amulic

**Affiliations:** 1 School of Cellular and Molecular Medicine, University of Bristol, Bristol, UK; 2 British Heart Foundation Cardiovascular Epidemiology Unit, Department of Public Health and Primary Care, University of Cambridge, Wort’s Causeway, Cambridge, UK; 3 Department of Human Genetics, The Wellcome Sanger Institute, Wellcome Genome Campus, Hinxton, Cambridge, UK; 4 The Francis Crick Institute, Antimicrobial Defence Laboratory, London, UK; 5 Cellular Microbiology, Max Planck Institute for Infection Biology, Chariteplatz 1, Berlin, Germany

**Keywords:** Biological sciences, Genetics, Immunology, Natural sciences

## Abstract

Neutrophils are essential immune cells loaded with cytosolic granules that contain potent antimicrobial and immunostimulatory molecules. Alterations of neutrophil granule contents are associated with immunodeficiency and hyperinflammation. Identification of regulators of granule development can aid in understanding of neutrophil-driven pathologies. Here, we perform a systematic prioritization of genetic variants associated with neutrophil cytometric side scatter (SSC), a proxy for granularity, identified in a genome-wide association study (GWAS) of blood parameters in healthy individuals. We show that triangulation of GWAS data with epigenetic and eQTL data identifies previously unknown factors regulating neutrophil granularity. We validate this approach using cellular and animal models to confirm that cyclin dependent kinase 6 (encoded by *CDK6*) regulates neutrophil granule development. CDK6 specifically regulates the abundance of primary granules without affecting neutrophil maturation. Our approach demonstrates the utility of cell counter-derived SSC data paired with genomics as a tool to investigate neutrophil development and function.

## Highlights

• GWAS reveals novel regulators of neutrophil granularity via SSC analysis
• CDK6 deficiency increases granule number and size in human and mouse neutrophils
• Neutrophil SSC is a robust proxy for granularity in genetic and functional studies

## Graphical abstract

**Figure:**
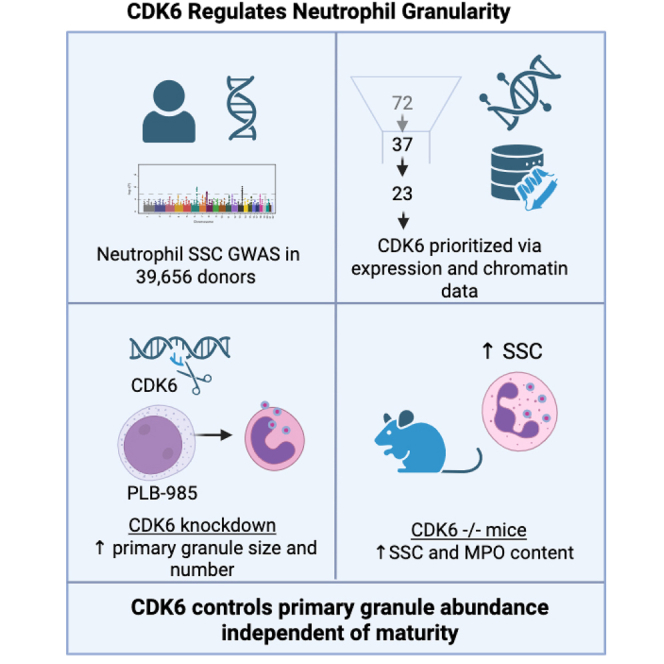

## Introduction

Cytosolic granules are a prominent morphological feature of neutrophils. They contain antimicrobial peptides, oxidant enzymes, proteases and siderophores.^1^ These contents can be mobilized by fusion with intracellular phagosomes, by extracellular release via granule exocytosis or by NETosis.^2^ Abnormal number or function of neutrophil cytosolic granules underlies multiple rare heritable and acquired immunodeficiency disorders.^3^^,^^4^ Neutrophil granule contents also contribute to sterile inflammation in common disorders such as atherothrombotic cardiovascular disease^5^ and cancer,^6^^,^^7^^,^^8^ and therefore represent an attractive drug target in inflammatory diseases.

Neutrophils generated from bone marrow precursors enter the circulation in a terminally differentiated state and have a low transcriptional profile compared to other immune cells. Transcriptomic studies of neutrophil granule formation are therefore challenging without bone marrow access. Assessing neutrophil granularity in different disease states can be performed manually using light microscopy of blood smears obtained from patient cohorts. However, quantitation of granules by microscopy is difficult to standardize and cannot be readily upscaled for studies in large populations. Granularity can also be detected using flow cytometry to measure the side scatter (SSC) of incident light as cells transit the cytometer analysis chamber.^9^^,^^10^ In contrast to light microscopy, this can be performed as a high throughput, automated assay as part of the complete blood count (CBC), which utilizes flow cytometry analysis to determine blood cell characteristics.^11^

Common genetic variants associated with multiple blood cell traits were recently reported as part of a comprehensive GWAS of 39,656 healthy volunteer blood donors enrolled to the INTERVAL study, in which cellular characteristics were measured using Sysmex SXN automated hematology analyzers.^12^^,^^13^ This identified many variants associated with neutrophil SSC as a surrogate for granularity. However, these variants have not yet undergone systematic annotation, prioritization, or validation to identify functionally relevant associations. Here, we report a detailed analysis of variants associated with neutrophil SSC, in which potential functional variants were first mapped to likely genes and then prioritized according to cellular expression patterns and triangulation against expressed quantitative trait (eQTL) and epigenetic localization data. We validated this analysis pipeline by performing a detailed functional analysis of *CDK6*, a neutrophil SSC-associated locus prioritized in our analysis but not previously recognized as a regulator of neutrophil granules.

## Results

### Prioritization of neutrophil SSC association signals

**Figure 1:**
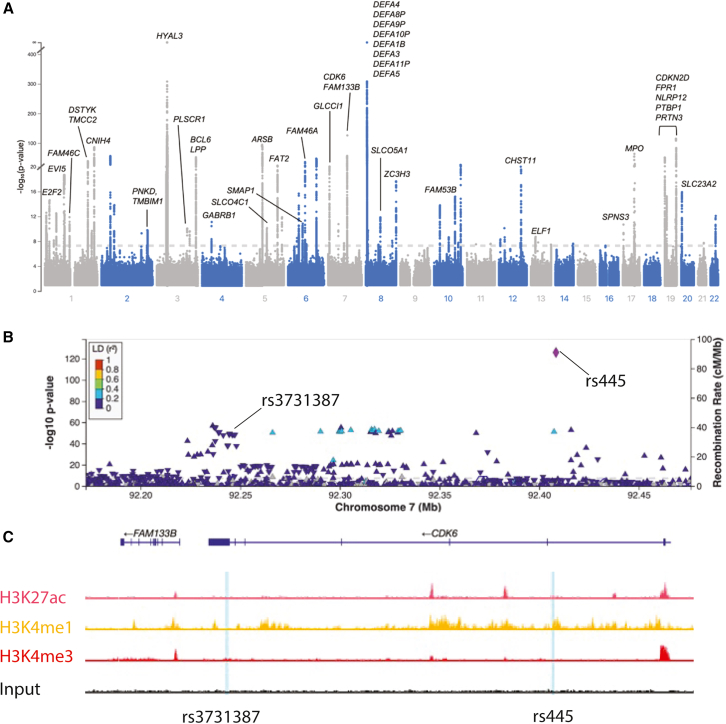
Genomic associations with neutrophil side scatter (A) Manhattan plot of GWAS SSC hits,^12^ with dotted line indicating *p*-value of <5 × 10^−8^ and x axis indicating chromosomal position. (B) A LocusZoom plot of the CDK6 locus with SSC-associated SNVs represented by the purple diamonds. LD = linkage disequilibrium, expressed as the square of the correlation coefficient (r^2^). (C) Epigenetic signature tracks from Blueprint database at the rs445 and rs3731387 (blue lines) SNV sites within the CDK6 gene.

GWAS analysis of blood cell parameters from healthy blood donors in the INTERVAL study identified 72 distinct (conditional *p*-value < 8.31 × 10^−9^) single nucleotide variants (SNVs) associated with neutrophil SSC (Figure 1A). Seventy-one of these SNVs showed no association with neutrophil count in the same population,^12^^,^^14^ indicating that SSC is a cellular characteristic with a distinct genetic architecture. Sixty of the conditionally significant neutrophil SSC variants were within 5kb of a protein coding gene enabling immediate assignment of a likely gene mediator using SNPNexus.^15^ For variants with multiple proximal genes, we used Variant Effect Predictor (VEP) to prioritize the single gene with most severe consequence.^16^ Of the 12 variants that were intergenic (>5kb from nearest gene), two were retained in the analysis because they colocalized with known whole blood quantitative trait loci (QTLs). The remaining 10 variants without an eQTL colocalization were not further analyzed (Figure S1).

The 62 filtered variants mapped to a total of 37 unique protein coding genes (Figure S1). Six of these had no evidence of expression in neutrophil progenitors in the Novershtern et al.^17^ dataset using the BloodSpot online resource^18^ or in mature neutrophils in NeutGX^19^ and were not considered further. Of the remaining genes, nine encoded proteins that were known components of neutrophil granules including myeloperoxidase (*MPO*), defensin alpha 4 (*DEFA4*) and proteinase 3 (*PRTN3*), providing strong evidence that the GWAS and analysis pipeline function as a tool for identifying biologically relevant genes. To further validate our approach, we selected MPO from these known granule proteins for genetically testing the effect on neutrophil granularity.

To discover unknown factors regulating neutrophil granules, we performed further prioritization (Figure S1) of the 23 mapped genes that were not already annotated granule components. Of these, 8 genes colocalized with the histone epigenetic modification markers H3K4me1, H3K27ac or H3K4me3 (indicative of an open chromatin state) in progenitors or mature neutrophils, identified in the Blueprint epigenome database.^20^ This yielded a prioritized gene shortlist of proximal variants, including ones linked with the transcription factor B-cell lymphoma 6 (BCL6), a solute transporter (SLC23A2), the GTPase-activating protein stromal membrane-associated protein 1 (SMAP1) and cyclin dependent kinase 6 (*CDK6*) (Table 1). These variants are likely to be functional because they lie within active chromatin regions in the relevant cell lineage (Figures 1B and 1C). *CDK6* was selected for validation among the prioritized unknown genes (Figure S1), as it had two distinct associated SNVs and the highest effect size.

**Table 1:** Prioritized shortlist of genes associated with regulation of neutrophil granularity

Gene name	Protein name: UniProt accession number	Known function	SNPs: association results (top 3) (rsID:GWAS catalog)
*CDK6*	Cyclin dependent kinase 6: Q00534	Cell cycle and transcriptional regulator (see discussion)	rs3731387, neutrophil side scatter; rs445; neutrophil count, monocyte count, myeloid white cell count
*CDKN2D*	p19/INK4d: P55273	Regulator of CDK6^21^	rs58573889; neutrophil side scatter
*E2F2*	E2F transcription factor 2: Q14209	Cell cycle regulator, in turn regulated by Rb, a target of CDK6^22^	rs2742972; red blood cell count, neutrophil side scatter
*PTBP1*	polypyrimidine tract binding protein 1: P26599	heterogeneous nuclear ribonucleoprotein, pre-mRNA processing	rs8103323; neutrophil side scatter
*BCL6*	B-cell lymphoma 6: P41182	Transcriptional repressor and proto-oncogene^23^	rs3774298; basophil percentage of white cell, neutrophil percentage of white cell, neutrophil forward scatter
*SMAP1*	Stromal membrane-associated protein 1: Q8IYB5	ARF6 GTPase-activating protein involved in clathrin-dependent endocytosis^24^	rs781984; neutrophil side scatter
*TMCC2*	transmembrane and coiled-coil domain family 2: O75069	Unknown, endoplasmic reticulum (ER)-residing transmembrane protein^25^	rs1172132; neutrophil side scatter
*SLC23A2*	solute carrier family 23 member 2: Q9UGH3	sodium-dependent vitamin C transporter^26^	rs6139587; neutrophil side scatter

### Knockout of MPO, a canonical primary granule component, alters neutrophil SSC

As an initial validation step, we analyzed neutrophil SSC in MPO −/− mice to ensure that deficiency of a known granule protein affects SSC. As expected from our GWAS result, MPO −/− neutrophils showed decreased neutrophil SSC, compared to wild-type (WT) (Figure S2; *p* = 0.0168, *n* = 7 per genotype).

### Selection of CDK6 as an exemplar regulator of neutrophil granularity

The filtered list of variants that were associated with neutrophil SSC included two intronic SNVs (rs445 and rs3731387) which had minor allele frequencies (MAF) of 0.1 and 0.24 and effect sizes of −0.12 and 0.28 respectively. These mapped to introns 2 and 7 respectively of *CDK6,* which encodes a kinase known to be expressed in myeloid cells (Table 1; Figures 1B and 1C). Moreover, both rs445 and rs3731387 colocalized with an H3K4me1 epigenetic signal present in neutrophils and metamyelocytes, but not other blood cells, suggesting the presence of an open chromatin state/transcriptional activity at this locus (Figure 1C). Interestingly, one of the other conditionally significant SNVs that was associated with neutrophil SSC (rs58573889) mapped to an intronic region of *CDKN2D*, which encodes p19INK4D, a direct binding partner and regulator of CDK6,^21^ further implicating the CDK6 complex in regulation of neutrophil granularity.

### Functional validation of CDK6 as a regulator of neutrophil granularity

**Figure 2:**
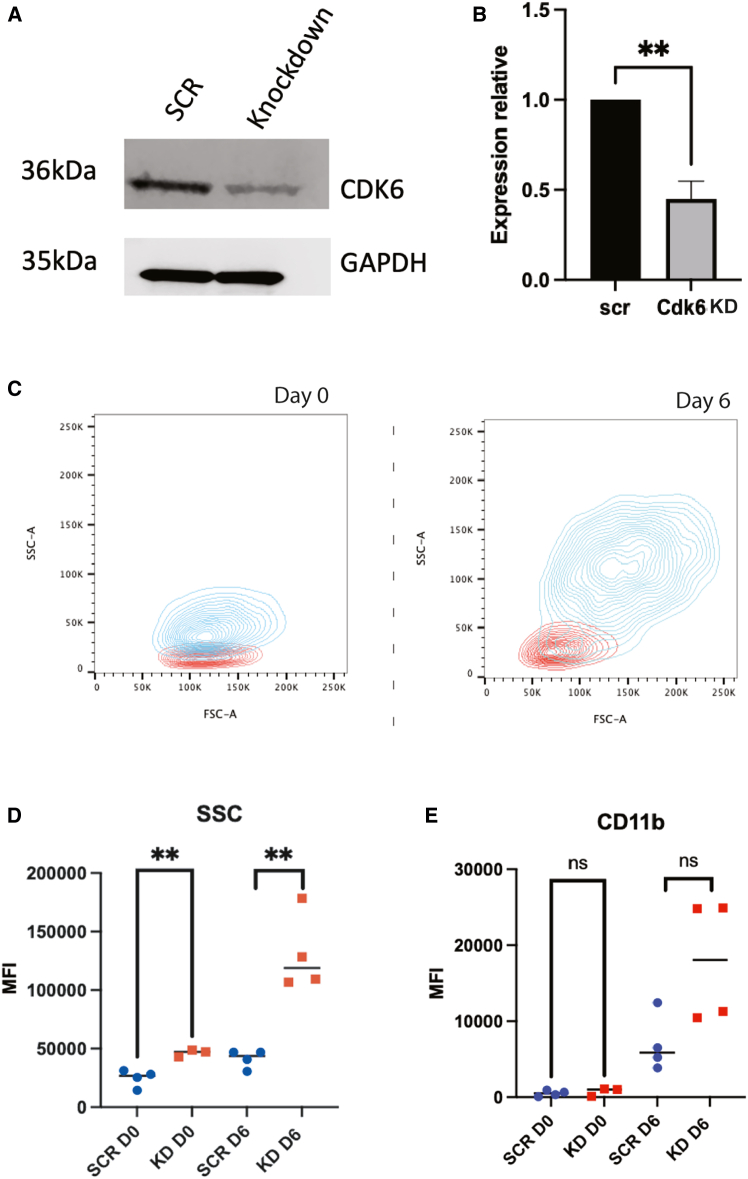
CDK6 regulates SSC in PLB-985 cells (A) Representative Western blot image showing CDK6 expression in SCR (scrambled control) and CDK6^KD^ PLB-985 cells, using GADPH as a loading control. (B) Quantification of CDK6 expression by densitometry of Western blots; CDK6 expression is normalized to expression of GAPDH (*n* = 3 experimental repeats). Data are represented as mean ± SEM. (C) Representative SSC and FSC FACS contour plot of SCR (red) and CDK6^KD^ (blue) PLB-985 cells at day 0 and 6 of differentiation. (D and E) (D) SSC and (E) median surface CD11b abundance quantified by FACS at day 0 (D0) and 6 (D6) of differentiation (*n* = 4 experimental repeats). Day 0: *p* = 0.53, Day 6: *p* = 0.051; F-test to compare variance not significant *p* = 0.53 & 0.24 respectively. Unpaired two-tailed t-tests. ∗∗∗∗*p* < 0.0001. ∗∗*p* < 0.005. ∗*p* < 0.05. MFI = mean fluorescence intensity.

To confirm the role of CDK6 in granule formation in human neutrophils, we used a human myeloid leukemia cell line (PLB-985), which can be differentiated into a neutrophil-like state.^27^ We knocked down CDK6 using a lentiviral construct delivering CRISPR/Cas9 with a guide RNA (gRNA) complementary to exon 4 of *CDK6*. We identified and expanded a clone with a 32-nucleotide deletion in the *CDK6* ORF (*CDK6*^KD^ cells), demonstrating approximately 50% reduction in CDK6 protein expression, compared to control cells targeted with a scrambled gRNA (Figures 2A and 2B), confirming partial knockdown.

We induced differentiation of PLB-985 cells to neutrophil-like cells with dimethylformamide (DMF). As expected, this was accompanied by an increase in both SSC and in surface expression of CD11b (Figures 2C–2E), indicative of maturation along the neutrophil lineage. Interestingly, *CDK6*^KD^ neutrophil-like cells had a significantly increased SSC compared to control cells, in both the progenitor and differentiated state (Figures 2C and 2D). These data indicate a negative relationship between CDK6 expression and neutrophil SSC, which reproduces the association observed in our GWAS analysis. In contrast, there was no difference in FSC, a surrogate of cell size, in the progenitor state, although a trend for increased FSC was observed after differentiation (Figure S3). We also observed a trend toward increased CD11b expression in *CDK6*^KD^ cells that did not reach statistical significance (Figure 2E) suggesting that the alteration in SSC was a consequence of increased cell granularity.

**Figure 3:**
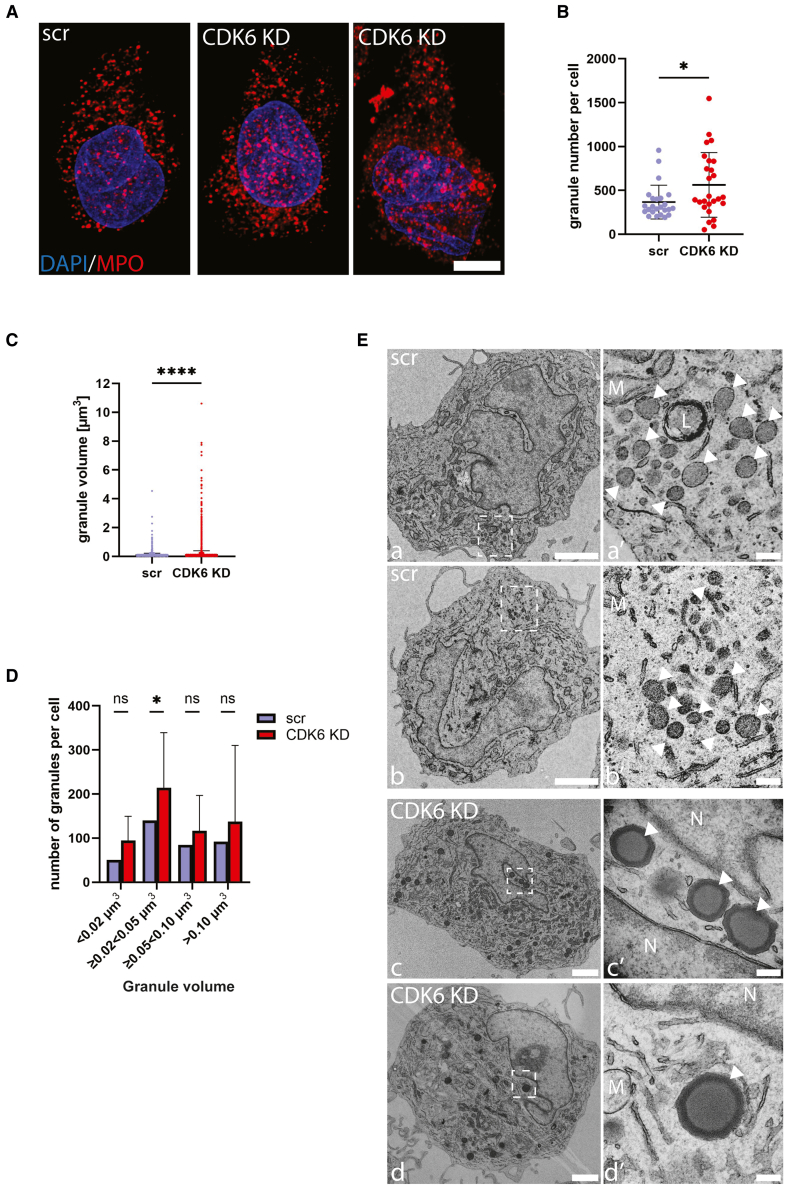
CDK6 regulates granule abundance and size in PLB-985 cells (A) Representative confocal images of primary granules in differentiated control (SCR) and CDK6 KD PLB-985 cells, stained with anti-MPO antibody. (B) Quantification of discrete MPO-positive granules from confocal images. (C and D) Volumetric quantifications (C) and distribution of granule volumes in size categories (D) of MPO-positive granules from confocal images; *n* = 3 stainings, unpaired t test (B and C) and two-way ANOVA (D), ∗*p* < 0.05, ∗∗∗∗*p* < 0.0001; data are represented as mean ± SEM. (E) Representative electron micrographs of SCR (wild-type) and CDK6KD differentiated PLB-985 cells; g: granule; m: mitochondria; N: nucleus. Arrow indicates site of higher magnification.

Increased SSC in CDK6^KD^ cells could result from changes in abundance of individual granules or from changes in the density of cargo in cells with an otherwise unaltered granule count. To distinguish between these two possibilities, we performed confocal immunofluorescence microscopy to enumerate granule frequency. Differentiated PLB-985 cells contain primary (azurophilic) granules, however, they lack secondary granules.^28^ We therefore stained cells with an anti-MPO antibody, which labels primary granules. We used the adaptive deconvolution method (Lightning mode) to obtain super-resolution images of MPO-positive granules (Figure 3A). We quantified the frequency of granules and found a significant increase in their abundance per individual CDK6^KD^ neutrophil-like cell, compared to individual SCR (WT) cell (Figure 3B). Furthermore, granule volume was also increased in CDK6^KD^ cells (Figures 3C and 3D). We confirmed elevated granule abundance using Sudan Black B dye staining, which can be used to non-specifically label neutrophil granules^29^ (Figure S4). We also used transmission electron microscopy (TEM) to visualize increased granule size (Figure 3E) and confirm increased frequency (Figures S5A and S5B). In summary, reduced CDK6 expression results in increased frequency and size of primary granules in human neutrophil-like cells.

### CDK6^−/−^ mice have increased abundance of primary granule proteins

**Figure 4:**
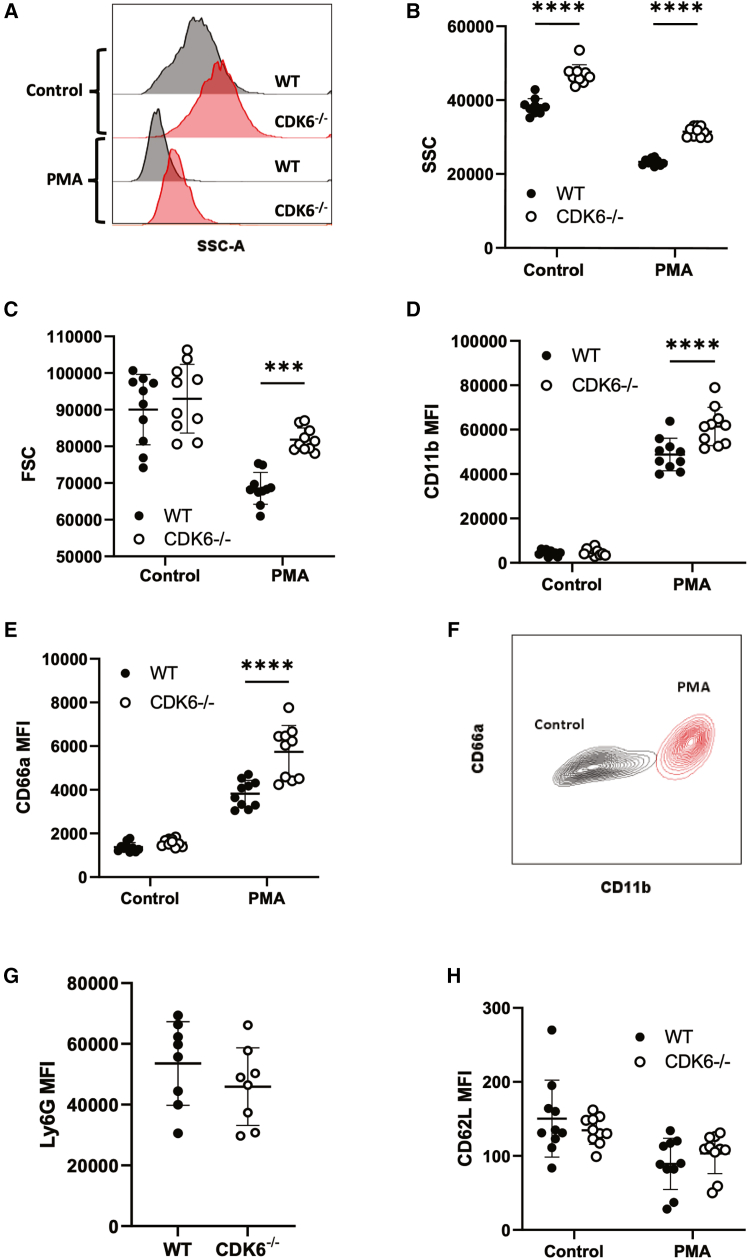
CDK6 regulates granularity in murine neutrophils (A and B) (A) Representative histogram and (B) quantification of SSC in PMA-stimulated (100 nM) and unstimulated (control) mouse marrow neutrophils, P = <0.0001. (C) FSC quantification in PMA stimulated and control CDK6^−/−^ and WT neutrophils, *p* = 0.5971 (control), *p* = 0.0004 (PMA). (D–H) MFI quantification, in PMA-stimulated and control mouse bone marrow neutrophils, of CD11b (D), *p* = 0.98 (control) and P= <0.0001 (PMA), CD66a (E), *p* = 0.747 (control) and P=<0.0001(PMA), Ly6G (G), *p* = 0.36 and CD62L (H), *p* = 0.546 (control) and *p* = 0.6109 (PMA). (F) An exemplar contour plot demonstrating PMA induced degranulation and activation. (B–E) and (G and H) *n* = 10 CDK6^−/−^ and 10 WT mice, grouped two-way ANOVA with Sidak’s multiple comparisons test, ∗∗∗∗*p* < 0.0001. ∗∗*p* < 0.005. ∗*p* < 0.05. MFI = mean fluorescence intensity. Data are represented as mean ± SEM.

To confirm the involvement of *CDK6* in granule formation *in vivo*, we studied neutrophils from a previously reported *CDK6*^−/−^ mouse model.^30^ Ly6G + neutrophils were identified in isolated bone marrow samples using the gating strategy described in Figure S6A. Neutrophil SSC was significantly increased in naive *CDK6*^−/−^ neutrophils compared with neutrophils from WT controls (Figures 4A and 4B), thereby reproducing the PLB-985 knockdown effect. As expected, stimulation with phorbol myristate acetate (PMA) to induce degranulation resulted in a reduction in SSC in both *CDK6*^−/−^ and WT cells but PMA-stimulated *CDK6*^−/−^ neutrophils retained higher SSC (Figures 4A and 4B). FSC was comparable in naive CDK6^−/−^ and WT neutrophils and was reduced in both genotypes following stimulation with PMA, reflecting change of cell shape upon activation (Figure 4C). CDK6^−/−^ neutrophils retained significantly higher SSC than WT neutrophils, even after correcting for FSC (Figure S3), suggesting additional differences in intracellular structure, beyond granule secretion, which seems intact in CDK6-deficient cells.

We next examined surface exposure of granule markers, as a readout of granule exocytosis. Granule markers in mice are not as well characterized as in humans. Surface expression of CD66a and CD11b, inferred from humans to be markers of secondary and tertiary granule exocytosis, respectively,^31^^,^^32^ increased in PMA-stimulated *CDK6*^−/−^ and WT neutrophils. Interestingly, despite starting with similar amounts, *CDK6* −/− neutrophils exposed significantly higher levels of both granule protein markers upon PMA stimulation (Figures 4D–4F), suggesting increased propensity for degranulation.

Since granularity increases during neutrophil maturation, the observed increase in SSC and granularity in the *CDK6* −/− neutrophils may reflect an indirect effect of *CDK6* on neutrophil maturation. In order to test this, we quantified surface expression of the neutrophil maturation markers Ly6G and CD101.^33^ There were no differences in Ly6G (Figure 4G) or CD101 (Figure S5C) expression between naive *CDK6*^−/−^ and WT neutrophils, suggesting comparable maturity. Similarly, cell surface expression of the neutrophil maturity and activation marker CD62L was similar in naive and stimulated CDK6^−/−^ and WT neutrophils (Figure 4H). Taken together, these data indicate that *CDK6* regulates SSC and granularity independently of maturation or activation.

**Figure 5:**
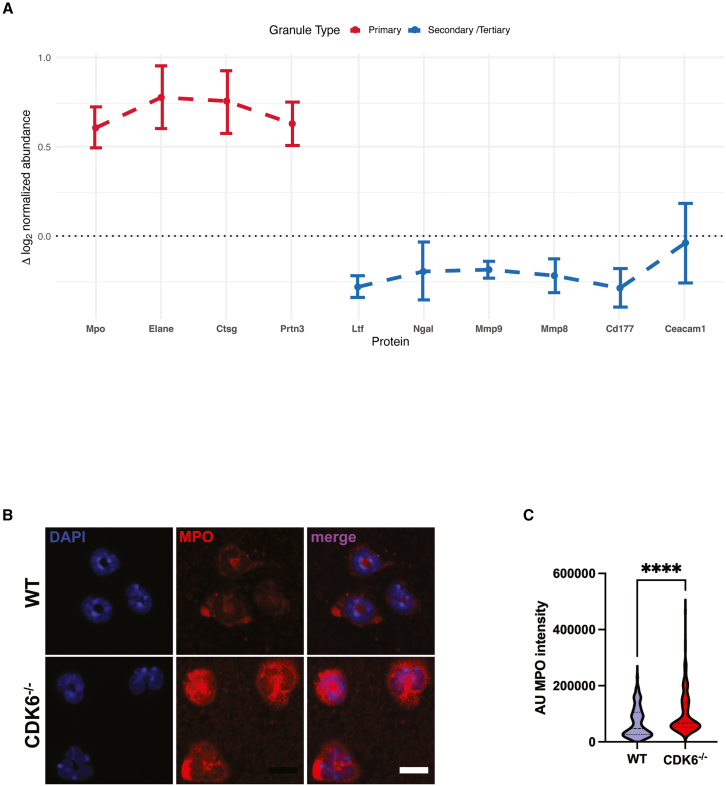
CDK6 regulates primary granules in murine neutrophils (A) Granule proteins displayed as log2 fold change in normalized abundance (*n* = 3 WT and *n* = 3 CDK6−/−). Granule proteins are classified as localized predominantly to primary (red) or secondary/tertiary (blue), based on inference from human data. Statistical analysis was performed by Welch’s *t* test, FDR adjusted (all nonsignificant). Data are represented as mean ± SEM. (B) Representative image of confocal immunofluorescence of MPO in WT and CDK6^−/−^ bone marrow neutrophils. Scale bar: 20 microns. (C) Quantification of MPO immunofluorescence in *n* = 3 mice per genotype, >70 cells per mouse assessed, AU = arbitrary units, unpaired T-test, ∗∗∗∗*p* < 0.0001.

In order to examine whether specific granule subsets are differentially expressed in *CDK6*^−/−^ neutrophils, we performed proteomic analyses using tandem mass tag spectrometry. A total of 871 proteins were significantly differentially expressed (*p*-value <0.05) in the naive *CDK6*^−/−^ neutrophils (*n* = 3), compared to WT controls (*n* = 3; Data S1). Principal component analysis showed homogeneity within genotype (Figure S7). Neutrophil granule contents are poorly annotated in mice, due to lack of granule fractionation studies. We therefore analyzed expression of peptides corresponding to well-established human granule markers. We found a consistent trend for elevated abundance of primary granule proteins in the *CDK6*^−/−^ neutrophils (Figure 5A), including neutrophil elastase, proteinase 3 and myeloperoxidase (MPO), although these differences failed to reach statistical significance after correction for multiple testing. On the other hand, proteins localizing to secondary and tertiary granules in humans, including lactoferrin, MMP8 and MMP9 did not show a trend for elevated expression (Figures 5A and S8). This suggested that the granule phenotype in the CDK6^−/−^ neutrophils was a result of dysregulated primary granule synthesis which occurs in promyelocyte stage.^34^ The proteomic result was validated by quantifying MPO abundance using confocal immunofluorescence imaging in isolated bone marrow neutrophils (Figure 5B). We confirmed increased cytoplasmic MPO content in the CDK6^−/−^ neutrophils (Figure 5C), in keeping with increased abundance of MPO positive granules in CDK6 KD PLB-985 cells.

## Discussion

Neutrophil granules contain specialized proteins with unique microbicidal and inflammatory properties. Regulation of granule cargo is central to determining the outcomes of infectious and inflammatory diseases. We refined a population-level approach for discovering previously unknown granule regulators, based on analysis of neutrophil SSC GWAS data. Our approach highlighted putative granule regulators, including multiple cell cycle proteins, the transcriptional regulator *BCL6*, a GTPase-activating protein (*SMAP1*) and a vitamin C transporter (*SLC23A2*), as well as proteins of unknown function.

Although SSC can be a readout for other intracellular structures,^35^ in neutrophils this is primarily a granule assessment parameter. Zimmerman et al. compared Neut-GI (a Sysmex cell counter-derived parameter of SSC) with trained technicians’ visual assessment of granule content by manual microscopy in 158 peripheral blood smears and showed a statistically significant correlation (r^2^ = 0.84, *p* < 0.0001).^9^^,^^10^ Furthermore, there are multiple reports of correlations between neutrophil SSC indices and excessive or ‘toxic’ granulation in sepsis^36^ or hypogranulation in myelodysplasia.^37^^,^^38^ These reports, along with the genetic validation of our approach described above, confirm that SSC is a valid and robust parameter for overall neutrophil granularity assessment.^36^^,^^37^^,^^38^

We used two genetic approaches to validate CDK6 as a regulator of primary granule proteins. Consistent with this finding, the peak of CDK6 transcription occurs at the promyelocyte stage,^39^ when primary granules are synthetized. CDK6 and its paralog CDK4 are cell cycle regulators that control the transition from G1 to S phase in proliferating cells, by phosphorylating the transcriptional regulator retinoblastoma protein (pRB).^40^ CDK6 and CDK4 are redundant and loss of a single paralog does not affect cell cycle progression, however, CDK4/6 double knockout mice are embryonic lethal.^41^ CDK6 has a known role in hematopoiesis, specifically in inducing exit from quiescence in hematopoietic stem cells and in controlling hematopoietic stem cell self-renewal under hematopoietic stress.^42^ CDK6 knock-in variants increase the risk of hematological malignancy.^43^ Despite this, hematopoiesis in CDK6^−/−^ mice is only subtly perturbed, with alterations largely limited to T lymphoid lineages,^41^ with no alteration in total neutrophil counts.^44^ Interestingly, a recently developed conditional CDK6 knockout demonstrated that that this protein has functions independent of its kinase activity, in transcriptional regulation of genes involved in differentiation and inflammation.^45^ Importantly, CDK6 appears to be a major regulator of innate immunity, since CDK6−/− mice have a deficiency in NET formation and demonstrate impaired survival when challenged with the fungal pathogen *Candida albicans* (*C. albicans*).^44^ Additionally, SNPs in CDK6 are associated with rheumatoid arthritis.^46^ Both of these observations may be related to the perturbed primary granule development described herein, but this requires additional investigation.

The mechanism by which CDK6 suppresses the expression of primary granule proteins remains unclear. CDK6 has been reported to modulate activity of transcription factors NF-κB, VEGF-A and RUNX1,^47^^,^^48^^,^^49^ all of which are implicated in myelopoiesis. This could be tested by analyzing granularity in “kinase dead” knock-in mice^50^ or by quantifying transcription of granule genes in the PLB-985 model. Other transcription factors involved in myelopoiesis noted to be differentially expressed in Cdk6 knockout neutrophils in our proteomics data (see Data S1) include Sp3, which has been shown to regulate the expression of BPI in primary granules,^51^ Upstream Transcription Factor 2 (Usf2) and GA-binding protein transcription factor alpha (GABPA), predicted by Harmonizome3^52^ to regulate expression of primary granule proteins MPO, ELANE, FUC1, GUSB, and HSPA8. CDK6 could also be regulating propensity to degranulate, as reported for the related cell cycle kinase CDK5.^53^ This is consistent with our finding that CDK6^−/−^ mouse neutrophils have higher externalization rates of the granule marker CD66a.^54^^,^^55^ Primary granules consists of two subsets, one of which has a higher propensity for extracellular cargo release, while the other engages in fusion with the phagosome^56^; it remains unclear whether CDK6 regulates abundance of both subsets. In summary, CDK6 may be regulating granule synthesis and mobilization in various ways and additional research is needed to understand how this contributes to immunity.

### Limitations of the study

Our prioritization workflow could be further improved by integration of multiple lines of evidence,^57^ as annotation of neutrophil genes improves. Another limitation is the use of the PLB-985 cell line, which are neoplastic cells with an abnormal morphology. Furthermore, they also have abnormal granule composition and do not express all cell surface markers in a manner typical of primary mature neutrophils.^28^^,^^58^^,^^59^ Although we confirm our results with primary mouse neutrophils, due to restrictions in cell abundance we used bone marrow rather than peripheral blood and we also acknowledge the potential biological difference between mouse and human granulopoiesis. Similarly, granule composition in mice is not as well characterized as in humans and some of our markers (e.g., CD66A) are based on inference from humans. A further limitation is that we used PMA as a stimulator of degranulation; this chemical has pleiotropic effects, and it will be important to test specific physiological inducers in the future. It also remains unclear how CDK6 deficiency leads to increased granule abundance and it will be important to clarify this in mechanistic studies.

Our validation of CDK6 as a regulator of neutrophil granule biology suggests that other novel hits from GWAS studies are worth investigating. Furthermore, it accentuates the wealth of relevant human data that can be obtained from clinical cell counters. Targeting CDK6 and the other candidate genes reported here may be a potential avenue for development of innovative therapies for inflammatory diseases associated with dysregulated neutrophil responses.

## Resource availability

### Lead contact

Dr Borko Amulic, School of Cellular and Molecular Medicine, University Walk, Bristol, BS8 1TD, United Kingdom. Email: borko.amulic@bristol.ac.uk.

### Materials availability

This study did not generate new unique reagents.

### Data and code availability

The following materials which support this publication are available for public access via GEO dataset: Series Accession: GSE119453. ID:200119453. This paper does not report original code. Any additional information required to reanalyze the data reported in this paper is available from the lead contact upon request.

## Acknowledgments

We thank all the members of the Zychlinsky lab, Max Planck Institute for Infection Biology, Berlin, Germany for laboratory access. We thank the staff at the University of Bristol flow cytometry (Dr Andy Herman, Poppy Miller, and Helen Rice) and proteomics (Dr Kate Heesom and Dr Philip Lewis) core facilities. We acknowledge Stephen Cross from the Wolfson Bioimaging Facility for assistance with image analysis.

This work in B.A.’s lab is funded by 10.13039/501100000265MRC grant MR/R02149X/1. K.F. was funded through an NIHR Academic Clinical Fellowship and by a GW4-CAT Wellcome trust Clinical PhD Fellowship. This work was supported by the 10.13039/100010438Francis Crick Institute, which receives its core funding from the 10.13039/501100000265UK Medical Research Council (FC0010129), 10.13039/501100000289Cancer Research UK (FC0010129), and the 10.13039/100010269Wellcome Trust (FC0010129). The views expressed are those of the authors and not necessarily of the NHS, the NIHR, the Department of Health, or Wellcome Trust.

## Author contributions

K.F. and K.B. designed and performed the GWAS variant filtering pipeline. P.A. conceptualized, performed and analyzed the GWAS. K.F., C.N., W.B., F.P.-G., C.J.H., S.G., P.Z., C.R., and D.C. designed and carried out experiments and analyzed data. B.A. and V.P. organized mouse breeding and provided mice. B.A. and A.M. conceived and supervised the study. K.F., A.M. and B.A. wrote the manuscript. All authors read, provided input, and approved the final manuscript.

## Declaration of interests

The authors declare no competing interests.

## STAR★Methods

### Key resources table

**Table:** 

REAGENT or RESOURCE	SOURCE	IDENTIFIER
**Antibodies**		

CD11b (human)	BioLegend	#101216; RRID:312799
Zombie/Aqua	BioLegend	#423101
CD66a	BioLegend	#134540
CD11b (mouse)	BioLegend	#101216; RRID:312799
CD62L	BioLegend	#104406; RRID:313093
Ly6G	BioLegend	#127628; RRID:2562567
CD45	BioLegend	#103132
CD101	Invitrogen	RRID:AB_1210728

**Software and algorithms**		

FlowJo (LLC)	10.8.1	Becton, Dickinson and Company; 2023.
Prism	9.5.0	GraphPad Software, San Diego, California USA, www.graphpad.com
Image Studio Lite	4.0	LI-COR Biosciences, Ltd - UK
Adobe Illustrator	27.7	Adobe Inc.
ImageJ (Fiji)	1.5R	Rasband W, NIH USA
RStudio	4.3.0	N/A

### Experimental model and study participant details

PLB-985 cell line (female, age at sampling 36), unauthenticated, tested negative for Mycoplasma.

Cdk6^−/−^ mice (B6.129S4-Cdk6^tm1^/J) were generated by a knock-in approach.^30^ MPO -/- (B6.129X1-*Mpo*^*tm1Lus*^/J) mice were previously described^60^ Animals were housed in approved specific pathogen free (SPF) conditions at the Max Planck Institute for Infection Biology or the Francis Crick Institute and maintained on a 12-hour light/dark cycle and fed *ad libitum*. A mix of male and female 8–12-week-old mice were used but the effect of sex on the results was not analysed.

### Method details

#### GWAS

The analyses of INTERVAL study data described in brief here were performed by Akbari et al. (University of Cambridge, UK).^13^

Associations between phenotypes and imputed allelic doses in 39,656 INTERVAL study participants were calculated using BOLT-LMM software which uses a linear mixed model. The top 10 principal components from the genetic relationship matrix and NHSBT donation centre were included as covariates. Significantly associated variants were identified using a stringent p-value threshold of 8.31 x10^-9^ to accommodate for low-frequency SNPs, as per preceding UK10K Consortium publications. The phenotypic variation explained per variant (R^2^) was calculated as 2·MAF·(1−MAF)·*β*2.

To identify independently associated variants, conditional association analyses were performed. In brief, the genome-wide significant variants were partitioned into blocks separated by 10Mb and a multivariable linear regression performed for each block, using the variant with the lowest p-value as a covariate in the model. Repeated iterations were performed until all variants were below the genome-wide significance threshold (8.31 x10^-9^), the blocks merged, and the analysis repeated to account for any residual long-range linkage disequilibrium (LD). The authors then used a standard LD (r^2^ >0.8) based greedy clumping algorithm to partition the conditionally significant associations across different traits into groups of correlated SNPs. All variants were annotated with rsIDs using dbSNP v14.^61^

Summary statistics for neutrophil SSC plus the results of conditional association analyses were made available to us for this study. Note than individual-level genotype data was not available. We performed an eQTL analysis using QTLbase to query SNPs that did not map to a nearby gene and selected those associated with an eQTL in blood tissue.^62^

#### Cell culture and differentiation

The PLB-985 cell line (a kind gift from Prof. Mary Dinauer, Washington University) was cultured as previously described.^44^ Cells were passaged twice a week to maintain a confluency of ∼1.5-2x10^6^ /ml. To initiate differentiation, the PLB-985 cells were seeded in a 6 well plate at 1x10^6^ cells per well in RPMI-1640 media supplemented with 2.5% FBS, 2 mM Glutamine, 100units/ml Penicillin, 100μg/ml Streptomycin, 0.5% N,N-dimethylformamide (DMF) (Sigma) and 1x Nutridoma-CS (50X) (Roche). The cells were collected from the wells on day 6 by centrifugation at 400g for 10 minutes and layered on top of 3ml of Histopaque-1077 (Sigma) and centrifuged at 800g for 20 min with soft deceleration. The top cell layer was collected, washed with 10 ml assay media, resuspended in 1ml phosphate-buffered solution (PBS) and counted using a haemocytometer.

#### Genome editing in PLB-985 cells

CRISPR/Cas9 genome editing was performed using a pLentiCRISPRV2 construct with a puromycin selection marker (GenScript), as described in Sollberger et al.^63^ Lentiviral particles were produced using psPAX and pMD2.G packaging plasmids in HEK293T cells. The gRNA targeting *CDK6* was TTAGATCGCGATGCACTACTCGG. Induced loss of function variants in single cell clones, were confirmed by sequencing using the Outknocker protocol.^64^

#### Analysis of cells by flow cytometry

For the PLB-985 cell line, 5x10^5^ cells were centrifuged in a U-bottom 96 well plate and washed with PBS. Following addition of 25 μl Fc Block, the cells were incubated on ice for 5 minutes before addition of 25 μl of antibody mix (anti-CD11b, anti-CD66a, 1:50 dilution in MACS buffer: PBS + 0.5% BSA +5 mM EDTA). Antibody manufacturer and concentration are shown in key resources table: Flow cytometry antibodies. The cell samples were then incubated again on ice in the dark for 30 minutes and washed twice with 100 μl MACS buffer before analysis on LSR Fortessa X20 (BD Biosciences) flow cytometer. Cells were gated for singlet live population.

For the murine cells, the contents of each culture well were resuspended in Fc block 25 μl (BD Pharmingen #553142) and then incubated on ice for 5 mins. Antibodies at concentrations indicated in key resources table were added to each well. Following a further incubation for 30 mins on ice in the dark, 200 μl MACS buffer was added to each well. After centrifugation at 400G for 5 mins and aspiration of the supernatant, the cell pellet was resuspended in 100 μl 4% paraformaldehyde (PFA) (Electron Microscopy Sciences) and incubated at room temperature in the dark for 20 minutes. The cells were then mixed with 100 μl of chilled MACS buffer, centrifuged again and resuspended in 200 μl MACS buffer. Protein expression levels were measured using an LSR Fortessa X20 flow cytometry (BD Biosciences) gated for single events. Neutrophils were selected as cells with dual positive CD45 and Ly6G staining.

#### Analysis of cells by electron microscopy (TEM) and Sudan Black B dye

For TEM, samples are prepared by manual fixing in 2.5% glutaraldehyde/formaldehyde, 2% PFA in 0.1 mol/L sodium cacodylate/HCl buffer, pH 7.2–7.4, 4°C overnight, then postfixed in 1% osmium tetroxide for 1 h, followed by 3 washes in sodium cacodylate and 3 washes in ddH_2_O. Samples were stained overnight at 4°C in 1% aqueous filtered uranyl acetate en-bloc with Durcupan™ resin embedding. 70 nm sectioning was with Reichert Ultracut S and imaging performed with Thermo Scientific Talos L120C 120 kV TEM.

For Sudan Black B staining, Sudan black 0.7 g was prepared in propylene glycol 85% and heated to 100°C with constant stirring. Cytospins of PLB-985 cells at Day 6 of differentiation were heat-fixed and flooded with dye for 6 mins, washed with 50% ethanol and rinsed in distilled water. Imaging was performed 100X lens with oil and Olympus BX50 light microscope.

#### Mouse breeding and isolation of bone marrow

Mouse breeding and experiments were approved by the Berlin state authority *Landesamt für Gesundheit und Soziales* (Germany) or the Home Office (UK). Neutrophils were isolated from bone marrow using Ly6G positive selection kit (Miltenyi Biotec), according to manufacturer’s instructions.

#### Mouse neutrophil degranulation assay

The mouse neutrophils were seeded in 200 μl prewarmed RPMI+2 mM L-Glutamine at 500,000 cells/well, in a U- bottomed 96 well plate and incubated at 37°C for 20 min, followed by stimulation with 100 μM PMA for 30 min. The cells were then centrifuged at 400xg and the supernatant was removed. Degranulation was analysed by flow cytometry as described above.

Data were analysed including compensation matrices on FlowJo (FlowJo LLC) and transferred to GraphPad Prism for statistical analysis. One-Way ANOVA statistical tests were performed between groups with either Tukey’s multiple comparisons testing or Two-Way grouped ANOVA with Šídák’s multiple comparisons testing.

#### Proteomic analysis

Protein extracts from the mouse neutrophils underwent TMT Labelling and analysis by high pH reversed-phase chromatography, as previously described (Rice et al. Life science alliance).^61^

The raw data files were processed and quantified using Proteome Discoverer software v2.4 (Thermo Scientific) and searched against the UniProt Mouse database (downloaded July 2021: 35859 entries) using the SEQUEST HT algorithm. Peptide precursor mass tolerance was set at 10 ppm, and MS/MS tolerance was set at 0.6Da. Search criteria included oxidation of methionine (+15.995Da), acetylation of the protein N-terminus (+42.011Da) and Methionine loss plus acetylation of the protein N-terminus (-89.03Da) as variable modifications and carbamidomethylation of cysteine (+57.0214) and the addition of the TMTpro mass tag (+304.207) to peptide N-termini and lysine as fixed modifications.

After analysis in Proteome Discoverer 2.1, the data were processed and further analysed in the R statistical computing environment. The protein groups were reassessed by an in-house script which selects a master protein firstly by ID and quantitation metrics, then by the annotation quality of uniprot accessions. Data were log_2_ transformed, and statistical significance was calculated using Welch’s *t* test. Heatmaps and barcharts were generated with Prism Software. Data was uploaded to FIGSHARE: https://figshare.com/articles/dataset/CDK6_271123_6Plex_TMT_csv/29382311?file=55567670.

#### MPO immunofluorescence microscopy

Mouse marrow neutrophils were seeded onto glass coverslips and fixed with 4% PFA. The cells were permeabilized with 0.3% triton-X for 5 minutes, washed with PBS and then coated with blocking buffer (pH 7.5 PBS, 0.05% Tween 20 (Sigma, 9005-64-5), 3% goat serum (Sigma, G9023), 3% gelatin from cold water fish skin (Sigma, G7041-100G), 1% BSA (Millipore Probumin, 81-065-1) for 1 hour at RT. The coverslips were incubated for 2 hours with 1:100 diluted anti-MPO antibody (DAKO, A0398), then with 1:500 diluted secondary Goat anti-Rabbit IgG (H+L) Cross-Adsorbed Secondary Antibody, Alexa Fluor™ 594 for 2 hours at RT. The coverslips were mounted with ProLong™ Gold antifade reagent with DAPI (Invitrogen, P36941). Imaging was performed using Leica DMI6000 inverted epifluorescence microscope and acquired with Photometrics Prime 95B sCMOS camera. Acquired images were analysed using Fiji.

For human PLB-985, procedure was as above, with the addition of a 0.01% saponin permeabilization step. MPO antibody (R&D systems; AF3667) was used at 1:20 dilution, for 1 hour at RT. Images of cytoplasmic granules in PLB-985 cells were acquired using a Leica SP8 AOBS confocal laser scanning microscope with a 63x/1.4NA oil objective with 5× pre-acquisition zoom, followed by LIGHTNING adaptive image restoration. For each condition, at least 21 whole cells were imaged, across three independent experiments. Images were analyzed using the Fiji Modular Image Analysis (MIA) plugin with a custom workflow. Briefly, granule positions within cells were identified using the TrackMate spot detector. Images were then binarized, granules were separated using watershed segmentation and their volumes were quantified.

### Quantification and statistical analysis

Performed as stated in figure legends using Prism GraphPad software. In all figures p < 0.05 (∗), p < 0.01 (∗∗), p < 0.001 (∗∗∗), p < 0.0001 (∗∗∗∗), ns = not significant. N= number of mice/cells or experiments performed as stated in figure legend. SEM refers to the standard error of the mean.

## Supplemental information

Document S1. Figures S1–S8

Data S1. Mouse Neutrophil Proteomics
