# Supplementary material for: Genome-wide association analysis of neutrophil granularity identifies CDK6 as a regulator of primary granules
Source: iScience. 2025 Jul 7;28(8):113072. doi: 10.1016/j.isci.2025.113072 (PMC12432455; doi:10.1016/j.isci.2025.113072)

iScience, Volume *28*

Supplemental information

Genome-wide association analysis of neutrophil granularity identifies CDK6 as a regulator of primary granules

Kathryn Fleming, Kate Burley, Fernando Ponce-Garcia, Parsa Akbari, Claire Naveh, Chris Rice, Przemysław Zakrzewski, Willem Gibbs, Sarah Groves, Drinalda Cela, Venizelos Papayannopoulos, Christopher J. Harbort, Andrew Mumford, and Borko Amulic

Supplementary Figure 1

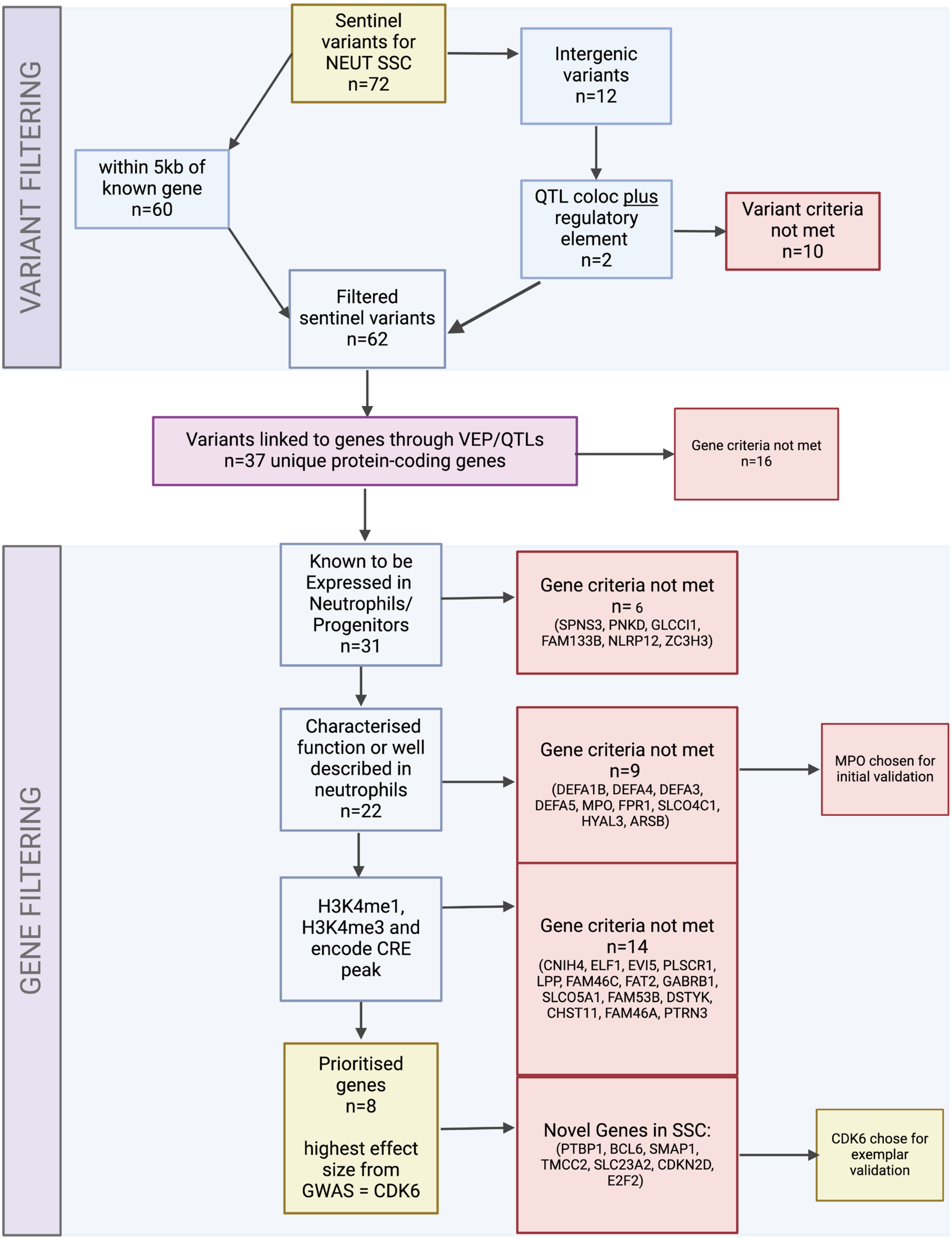

Created in https://Biorender.com

Supplementary Figure 2

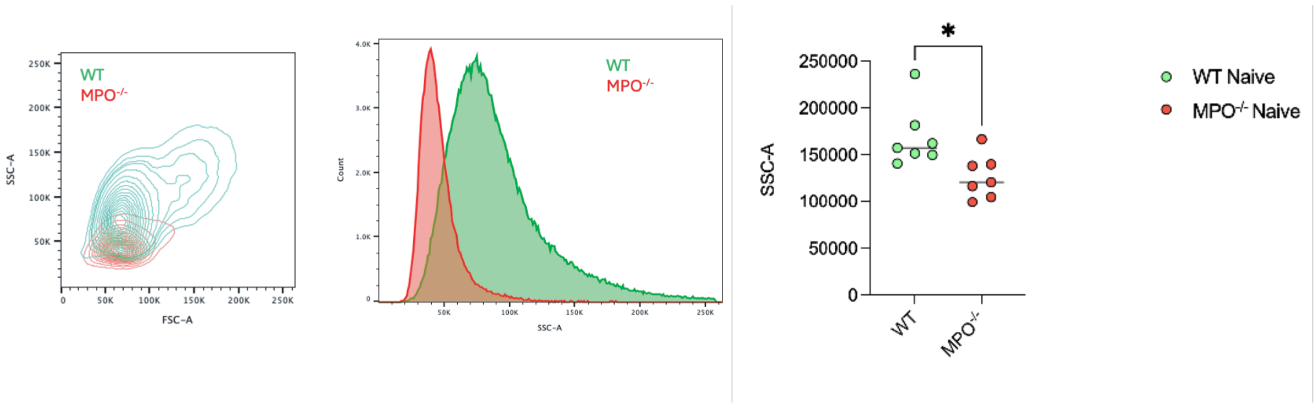

Supplementary Figure 3

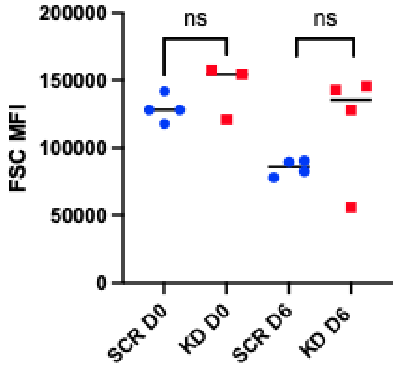

Supplementary Figure 4

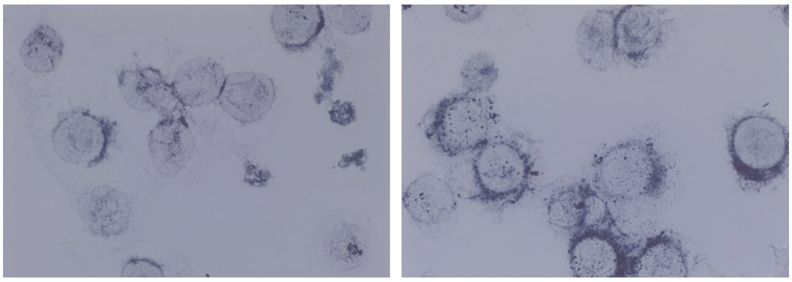

PLB-985 SCR D6

PLB-985 CDK6KD D6

Supplementary Figure 5

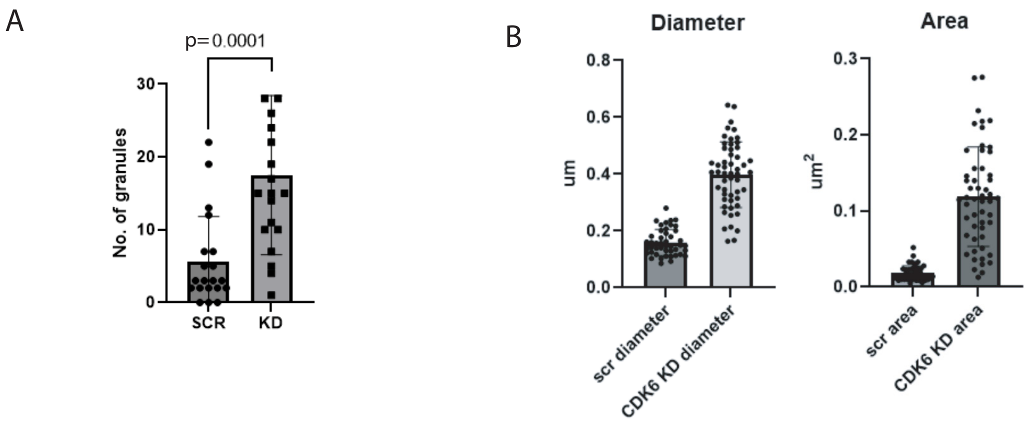

Supplementary Figure 6

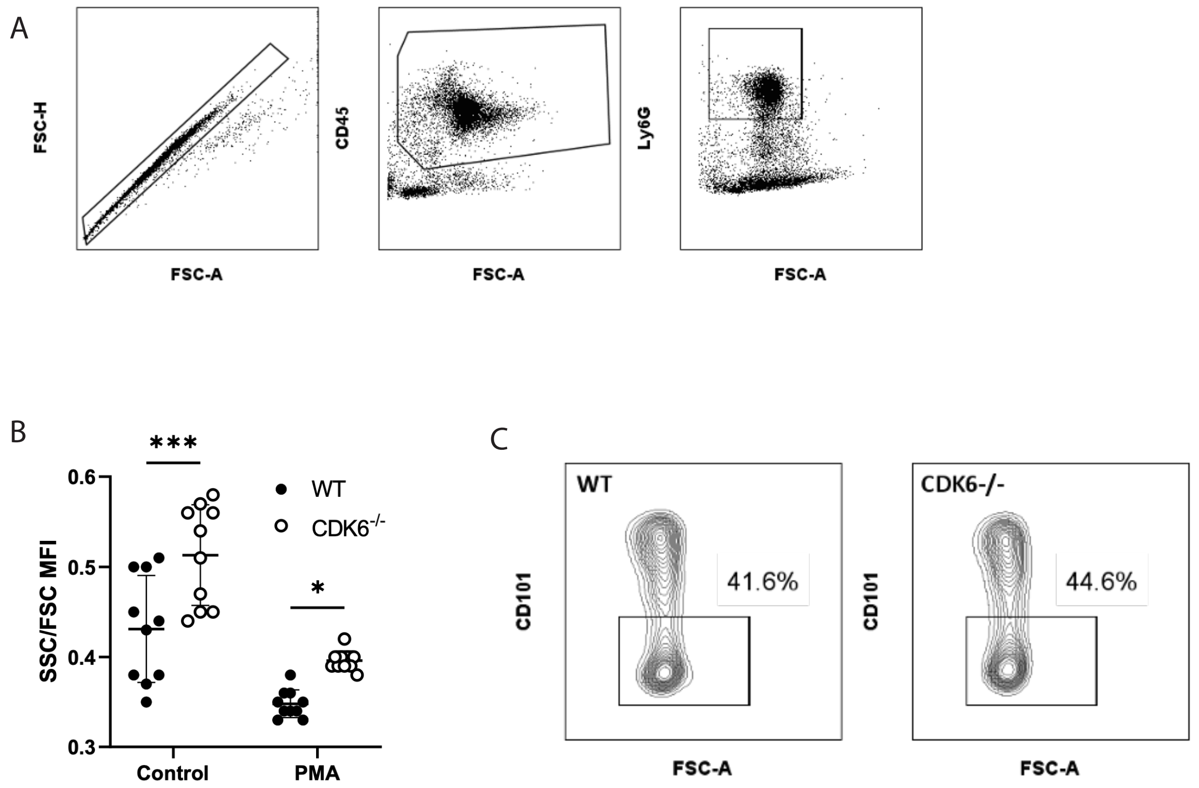

Supplementary Figure 7

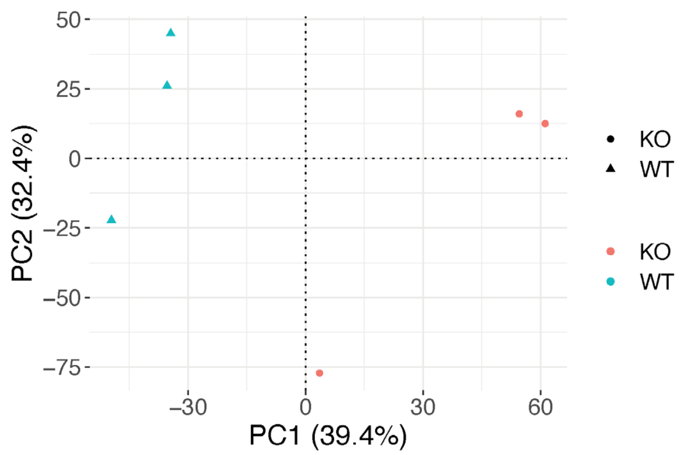

Supplementary Figure 8

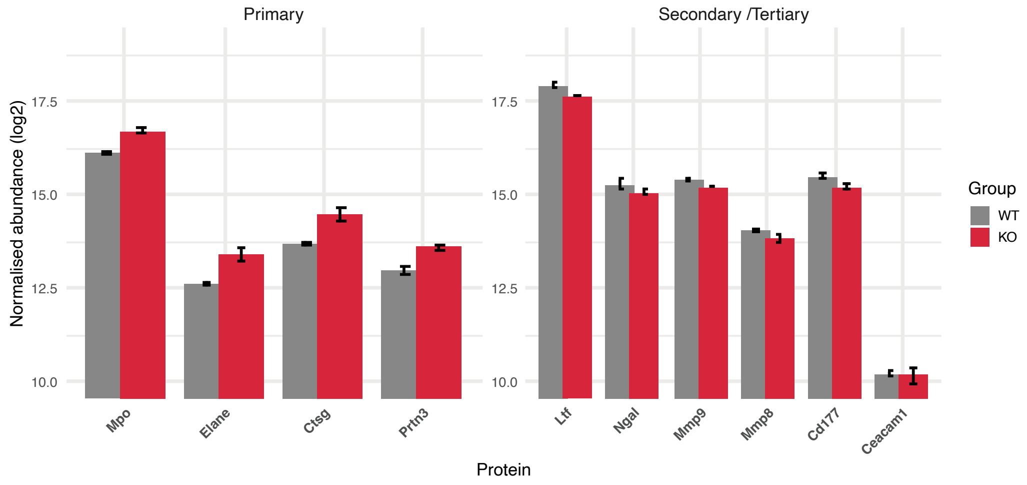

Supplement: Document S1. Figures S1–S8 [file mmc1.pdf]
